# Therapeutic Induction of Tertiary Lymphoid Structures in Cancer Through Stromal Remodeling

**DOI:** 10.3389/fimmu.2021.674375

**Published:** 2021-05-27

**Authors:** Anna Johansson-Percival, Ruth Ganss

**Affiliations:** ^1^ Cancer Microenvironment Laboratory, Harry Perkins Institute of Medical Research, QEII Medical Centre, Nedlands, WA, Australia; ^2^ Centre for Medical Research, The University of Western Australia, Crawley, WA, Australia

**Keywords:** light, LTβR, tumor, TLS, ICB, vascular normalization

## Abstract

Improving the effectiveness of anti-cancer immunotherapy remains a major clinical challenge. Cytotoxic T cell infiltration is crucial for immune-mediated tumor rejection, however, the suppressive tumor microenvironment impedes their recruitment, activation, maturation and function. Nevertheless, solid tumors can harbor specialized lymph node vasculature and immune cell clusters that are organized into tertiary lymphoid structures (TLS). These TLS support naïve T cell infiltration and intratumoral priming. In many human cancers, their presence is a positive prognostic factor, and importantly, predictive for responsiveness to immune checkpoint blockade. Thus, therapeutic induction of TLS is an attractive concept to boost anti-cancer immunotherapy. However, our understanding of how cancer-associated TLS could be initiated is rudimentary. Exciting new reagents which induce TLS in preclinical cancer models provide mechanistic insights into the exquisite stromal orchestration of TLS formation, a process often associated with a more functional or “normalized” tumor vasculature and fueled by LIGHT/LTα/LTβ, TNFα and CC/CXC chemokine signaling. These emerging insights provide innovative opportunities to induce and shape TLS in the tumor microenvironment to improve immunotherapies.

## Introduction

Unprecedented success of immune checkpoint blockade (ICB) in melanoma patients has sparked considerable interest in immunotherapies (1). Treatment with immune modulatory antibodies has also highlighted the critical importance of an immune “hot” tumor environment for therapeutic responsiveness (2). Considerable efforts are now being directed into increasing responsiveness to ICB in all cancer patients.

The tumor microenvironment including stromal innate immune cells, fibroblasts and the vasculature has become a major target for new therapies aiming to increase intratumoral T cell numbers and their activation status prior to ICB (3, 4). Spontaneous and/or therapeutic increase of T cell numbers into tumors can result in the formation of TLS (3, 5). These TLS have the ability to effectively prime naïve T cells entering through high endothelia venules (HEV) (6). Notably, the presence of TLS predicts and improves efficacy of immunotherapy in mice and humans (7).

In this review, we delineate common features of peripheral lymph nodes (LNs), inflammation- and cancer-associated TLS, and discuss the relationship between the presence of TLS, lymphocyte priming and response to immunotherapy. We further elaborate on potential drivers for intratumoral TLS formation and how TLS could be exploited therapeutically, in particular for non-responsive, immune “cold” cancers.

## The Beginning: Development of Lymphoid Tissue

The immune system is comprised of organs and cell types that protect the host from foreign pathogens and disease. The highly specialized adaptive immune system consists of T and B lymphocytes that form in the bone marrow and later reside in secondary lymphoid organs (SLOs). SLOs are strategically placed to facilitate immune surveillance and priming of naïve T cells and also include LNs (8). The structural framework of LNs are fibroblastic reticular cells (FRCs) which mediate cross-talk between various immune cell populations throughout the LN. In addition, follicular dendritic cells (FDCs) that reside within B cell zones maximize interactions between antigens, antigen presenting cells and naïve lymphocytes (9). Embedded in the paracortical region of LNs are HEVs, highly specialized post capillary venules that serve as entry portals for naïve and central memory lymphocytes from the blood; this migration process is mediated by interactions of L-selectin expressed on lymphocytes and peripheral node addressins (PNAds) on HEVs (10). TLS are lymphoid aggregates similar to SLOs which develop in non-lymphoid tissue, for instance at sites of chronic inflammation (11). TLS vary in composition and maturity but share with SLOs separated B and T cell zones, stromal cells, and HEVs.

One proposed mechanism for the initiation of LN development is upregulation of chemokine (C-X-C motif) ligand 13 (Cxcl13) by lymphotoxin beta receptor (LTβR) expressing mesenchymal precursors known as lymphoid tissue organizer (LTo) cells (12). Cxcl13 subsequently attracts hematopoietic precursors or lymphoid tissue inducer (LTi) cells resulting in the first cluster of LTi cells and the initiation of LN development (12). Mature LTi express lymphotoxin alpha 1 beta 2 (LTα_1_ß_2_) which binds LTβR in activated LTo, resulting in further LTo maturation and expression of intercellular adhesion molecule 1 (ICAM1), vascular cell adhesion molecule 1 (VCAM1), chemokine (C-C motif) ligand 19 (Ccl19) and 21 (Ccl21), and Cxcl13 which recruit more LTi and promote interactions between LTi and LTo (8, 9). Mouse LTo may give rise to stromal lineages such as FRCs, FDCs, lymphatic endothelium and vascular endothelium within adult LNs (13).

Emerging evidence also highlights a crucial role of vascular endothelium in the development of LNs. In adult LNs, endothelial cells (ECs) and lymphatic endothelial cells (LECs) express LTβR; EC-specific deletion of LTβR by crossing vascular endothelial cadherin (VE-Cad)-Cre and LTβR^fl/fl^ mice results in compromised LN development with a reduced HEV network demonstrating the importance of EC-specific LTβR for HEV development and lymphocyte trafficking (14). Moreover, EC and to a lesser extent LEC-specific deletion of NFκB-inducing kinase (NIK), one of the major pathways downstream of LTβR signaling, results in an almost complete loss of peripheral LNs (15). In the remaining LN anlagen of these mice, CD4^+^ LTi cells are drastically reduced coinciding with very low VCAM1, ICAM1, Cxcl13 and Ccl19 expression levels suggesting that failure of LTi to engage with ECs during LN development prevents LTo activation. Furthermore, forced retention of LTi following treatment of pregnant mice with the drug FTY720 which sequesters lymphocytes in LNs, results in formation of mature ectopic LNs in the inguinal fat pad of the progeny (15). These findings imply that the numbers of LTi retained by EC/LECs may be an additional determinant of LN development, alongside interactions between LTi and mesenchymal LTo (16).

## TLS Form Under Inflammatory Conditions in Mice

**Figure 1:**
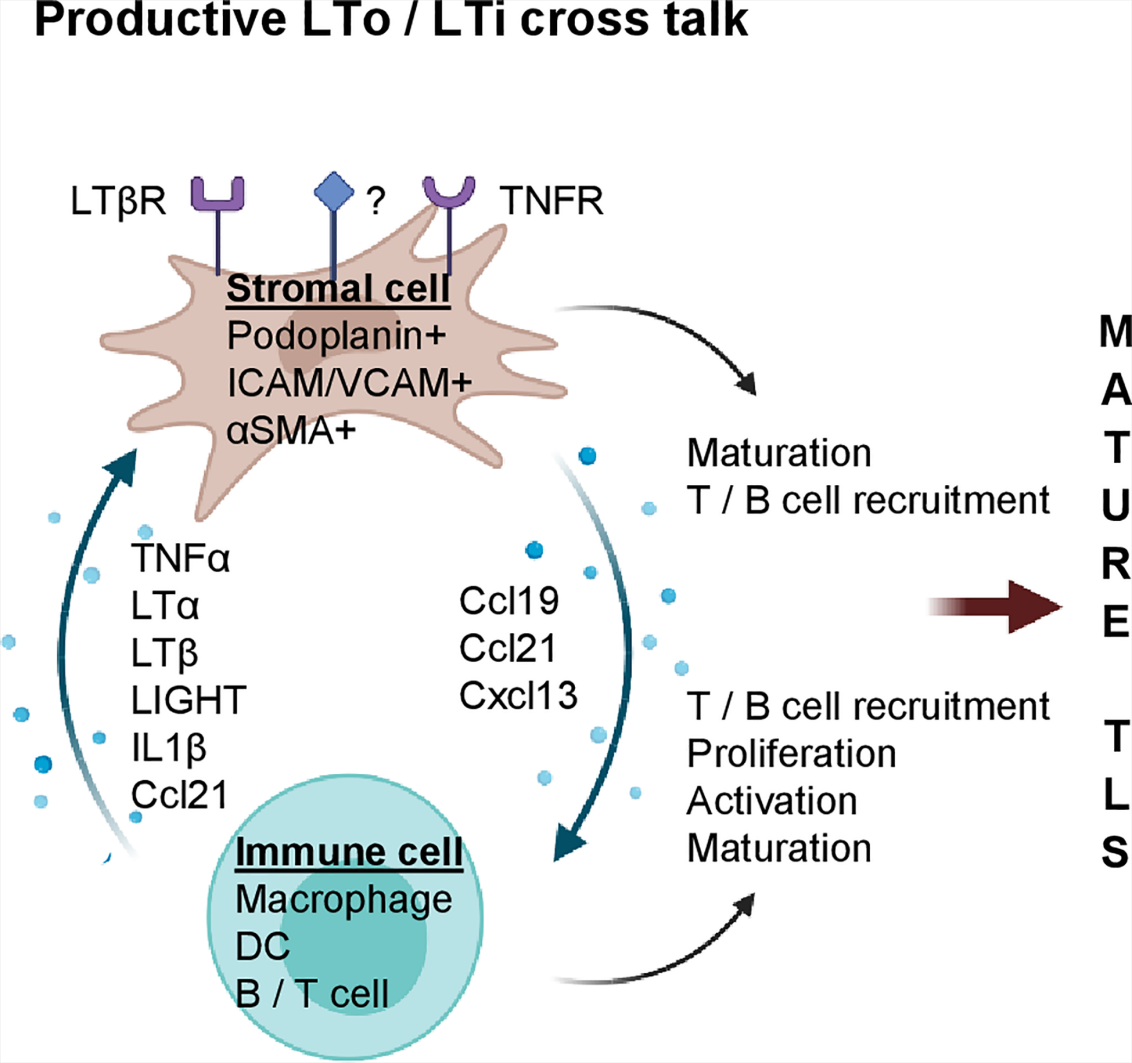
Stromal and immune cell cross talk mediate TLS formation during chronic inflammation. Potential cytokines/chemokines involved in immune (LTi) and stromal cell (LTo) cross-talk. Stromal cells express cytokine receptors such as LTβR and TNFR (and potentially others, marked with “?”); upon activation, LN inducing chemokines such as Ccl19, Ccl21 and Cxcl13 are secreted by stromal cells which increase immune cell density and foster their own maturation. Activated stroma and immune cells coordinate formation of LN aggregates which can mature into clusters containing T cells, B cells, FDCs and MECA79^+^ HEVs (mature TLS). Created with BioRender.com.

Although the initial events of LN development are not fully resolved, LTβR signaling is crucial for subsequent LN maturation, and also plays a major role in TLS formation during chronic inflammation in mice (**Figure 1**). For instance, in apolipoprotein E (ApoE)^-/-^ mice, LTβR expressing aortic smooth muscle cells (SMC) over time become activated and produce TLS inducing cytokines such as Cxcl13, Ccl21 and LTβ (17). This leads to the formation of mature aortic TLS containing B cell follicles and germinal centers (GCs), T cells and HEVs. Importantly, TLS assembly can be prevented by blocking LTβR signaling *in vivo* (17).

LTβR binds two ligands, the developmentally important LN-inducing cytokine LTα_1_ß_2_ and tumor necrosis factor superfamily (TNFSF) 14 or LIGHT. Increased LIGHT expression coincides with TLS formation in the pancreas of aged non-obese diabetic (NOD) mice; *in vivo* inhibition of LTβR prevents TLS formation and diabetes (18). TLS in mouse pancreatic islets can also be induced by overexpressing C-X-C chemokine receptor type 5 (Cxcr5), the receptor for Cxcl13 (19), Cxcl12, Ccl19 or Ccl21 (20) under the control of the rat insulin gene promotor. Interestingly, LTβR or LTα1β2 blockade prevents TLS formation in chemokine overexpressing mice (19, 20), implying that LTα1β2 and/or LIGHT are *bona fide* TLS inducers under inflammatory conditions. However, mechanisms leading to inflammation-associated TLS formation are complex and can involve a network of multiple immune and stromal cell types, and - besides LTα_1_β_2_ – other cytokines such as tumor necrosis factor alpha (TNFα), IL6, IL13, IL17, IL22 and IL23 (21–25).

In mouse inflammatory lesions, stromal cells can function as LTo by upregulation of the FRC markers podoplanin, Ccl19, Ccl21 and Cxcl13 which in turn stimulate lymphocyte recruitment to sites of inflammation (26, 27). For instance, in patients with primary Sjögren’s syndrome (pSS) and a mouse model of salivary gland inflammation, IL13 production by activated fibroblast activation protein (FAP)^+^ podoplanin^+^ fibroblasts, termed “immunofibroblasts”, is the earliest detectable event during TLS neogenesis which precedes lymphocyte recruitment into tissue and subsequent IL22/LTα_1_β_2_ secretion (24). As demonstrated in mice deficient for IL13 or its receptor IL4R, “immunofibroblast” activation is dependent on IL13/IL4R signaling and precedes their expansion which is subsequently regulated by lymphocyte-derived IL22 (28). Furthermore, genetic deletion of FAP^+^ fibroblasts abolishes TLS formation highlighting the LTo role of fibroblasts during TLS formation (24).

During ear inflammation in mice, induction of podoplanin^+^ stromal cells is dependent on myeloid cells, since depletion of CD11^+^ Gr1^+^ cells using monoclonal antibodies significantly reduces podoplanin^+^ cells (26). This suggests that circulating monocytes can acquire a postnatal role as LTi. Indeed, myeloid cells have been implicated in the development of TLS in various experimental systems. For instance, global overexpression of TNFα in mice by expressing a stabilized TNFα mRNA (TNF^ΔARE^) leads to the development of TLS in the intestine in a process which is dependent on F4/80^+^ myeloid cells (21). Mechanistically, F4/80^+^CD11b^+^ myeloid cells in the LN anlagen are the major source of TNFα and inducers of stromal maturation and expression of LTo chemokines such as Cxcl13, Ccl19 and Ccl21. The potency of these myeloid cells was further demonstrated by surgical transplantation of LN anlagen from TNF/RORc(γt)^-/-^ mice under the kidney capsule of RORC(γt)^-/-^ mice that lack classical LTi; this leads to LN development in the majority of mice thus demonstrating that TNFα producing myeloid cells have the capacity to induce LN formation (21). In atherosclerosis, M1-polarized macrophages act as LTi cells and produce high levels of LN-inducing cytokines such as TNFα and LTα (29). *In vitro* stimulation of vascular SMCs (vSMC) with M1 macrophage conditioned media induces an LTo profile and triggers the formation of TLS *in vivo* following vSMC injection (29). VSMC activation is dependent on TNFR signaling as blockade of TNFR1/2 *in vivo* abolishes the LTo phenotype and prevents TLS formation. Similarly, adipose tissue-associated TLS formation is dependent on myeloid derived TNFα and stromal expression of TNFR, but independent of LTβR signaling (27).

The effects of DCs on lymph angiogenesis and TLS induction have also been studied in multiple models (30–35). For instance, in a mouse model of atopic dermatitis, CD11c^+^ DCs accumulate around newly formed HEVs; inhibition of LTβR signaling or depletion of CD11c^+^ cells inhibits HEV formation (33). Similarly, following influenza virus infection in mice, lung CD11c^+^ DCs express TLS-inducing cytokines such as LTβ, Cxcl13, Ccl19 and Ccl21 which correlates with formation of mature TLS; *in vivo* depletion of CD11c^+^ cells or inhibition of LTβR signaling perturbs TLS formation (34). Moreover, in plaques arising in ApoE^-/-^ mice, LTβ producing CD11c^+^ CD68^+^ Ly6C^lo^ monocytes reside in close proximity to vSMCs and induce Cxcl13 and Ccl21 secretion, indicating a potential role of DCs as LTi (17). Overall, multiple models of chronic inflammation show that stromal cells can gain LTo function whilst inflammatory myeloid cells play a crucial role as LTi. Moreover, in the process of TLS formation, TNFα and LTβ serve important non-redundant roles.

## Spontaneous TLS Formation in Human Cancer

Tumors are described as “wounds that never heal” (36), and indeed rely on continuous stromal remodeling, inflammation and angiogenesis to support the rapidly growing cancer. The abnormal angiogenic tumor vasculature often lacks adhesion molecules such as ICAM/VCAM which prevents efficient lymphocyte-EC binding (37, 38). However, despite this “anergic” tumor vasculature, the tumor microenvironment (TME) can support naïve T cell infiltration, and spontaneous intratumoral TLS formation has been observed in a subset of patients across cancer types (7).

Although the precise mechanism of spontaneous TLS formation in human cancers is unknown, the presence of intratumoral TLS structures is often associated with a favorable clinical outcome and extended disease-free survival (7, 39–47). In hepatocellular carcinoma (HCC) for instance, the presence of intratumoral TLS reduces the risk for early relapse following tumor resection (43). In addition, mature TLS harboring GCs rather than poorly defined lymphocyte aggregates have the lowest recurrence risk (43). In human breast cancer, the presence of HEVs correlates with overall T and B cell infiltration, and improved prognosis (44, 45). Moreover, flow cytometry and gene expression analysis of CD4^+^ T cell subsets revealed that highly infiltrated breast cancers also harbor TLS, and express markers such as Cxcl13, ICOS, IFNγ and TBX21/T-bet, commonly associated with follicular T helper (T_fh_) and Th1 profiles (39, 40). In multiple human cancers such as lung, breast, pancreatic, gastric cancers and melanoma, TLS^high^ tumors harbor more activated, cytotoxic or naïve CD8^+^ T cells together with CD4^+^ T cells which are skewed to a Th1 and/or Th17 phenotype when compared to TLS^low^ tumors (41, 42, 44–47).

The presence of intratumoral TLS can be determined by analyzing chemokine gene-expression signatures which were first described in colorectal cancer (48) and subsequently validated for other types of cancer such as HCC, breast cancer and melanoma (43, 49, 50). The ability to assess TLS status prior to therapy is of clinical significance and may offer an opportunity to improve immunotherapy (49).

However, the predictive value of TLS for patient outcome is complex, and other parameters besides presence or absence of lymphocyte aggregates seem to be important. In colorectal cancer, for instance, TLS structures with high densities of M2 macrophages and T helper cells expressing GATA3, a master regulator of Th2 differentiation, contribute to immune suppression and thus correlate with relapse rather than improved prognosis (51). In HCC, TLS in the tumor margin are associated with an increased risk of recurrence (52). Moreover, TLS which arise in HCC patients, or mice with persistent and high NFκB activation in hepatocytes, promote tumor progression rather than anti-tumor immunity (52). Similarly, early human hepatic lesions can harbor immature TLS characterized by the expression of immune suppressive cytokines and T cell exhaustion markers such as IL10RA, TGFβ1, TIM-3 and PD-L2 (53). In other cancer types, for instance breast, colorectal and pancreatic cancers, TLS are often found in peri-tumoral locations, and are associated with more advanced disease (41, 54, 55). Overall, these studies indicate that intratumoral location and TLS maturity are crucial parameters for productive anti-tumor immunity and improved patient outcome (7, 56).

## TLS as Intratumoral Priming Sites for Adaptive Immunity

It is commonly accepted that naïve lymphocytes do not enter peripheral tissues or tumors, but circulate through lymphoid organs to encounter cognate antigen for activation. However, there is emerging evidence that HEV^+^ TLS may activate effector T cells intratumorally thus bypassing the need for tumor-antigen presentation in draining LNs (57). For instance, LIGHT accelerates development of diabetes in NOD mice even after surgical removal of pancreatic draining LNs implying that naïve T cells are primed within TLS in pancreatic islets (18). In B16 melanoma-bearing mice, adoptively transferred naïve anti-tumor T cells differentiate into effector cells, reduce tumor growth and improve survival even when lymphocyte egress from LNs is blocked by FTY720 (6, 58). This suggests that HEV^+^ mouse melanomas can facilitate naïve T cell infiltration, and support subsequent priming and differentiation (6).

Naïve T cell activation in TLS relies on the presence of antigen presenting cells such as B cells and DCs. Indeed, in lung (42, 59), breast (60) and renal cancers (61), a high density of TLS-associated mature DCs correlates with the degree of Th1 effector T cell infiltration and improved prognosis. Interestingly, DCs are also involved in HEV function. In peripheral LNs, for instance, DCs maintain HEV maturity and thus naïve T cell infiltration through LTβR signaling (30). In human breast cancer, DCs produce high levels of LTβ and the density of mature DC-LAMP^+^ DCs strongly correlates with the frequency of HEVs (60). Collectively, this indicates that DCs maintain HEV maturity and facilitate T cell egress and priming in both LNs and TLS.

B cells are an integral part of mature TLS and potent antigen presenting cells. In some cancers, B cells have been shown to foster tumor development by secreting factors which contribute to a pro-tumorigenic immune environment (62). However, mature B cells in TLS produce antibodies within GCs which correlates with a higher degree of T cell infiltration and disease free survival (63–65). Improved prognosis in human breast cancer is associated with CD4^+^ T_fh_ cells which produce an abundance of Cxcl13 and support B cell differentiation, TLS formation and GC maturation (39, 66). In pancreatic adenocarcinoma (PDAC), the presence of B cells within mature TLS correlates with improved prognosis in patients, or increased immune response to vaccination in mice (67). Furthermore, initial evidence in human melanoma suggested a potential link between antibody producing B cells and ICB responsiveness (68, 69). This has now been confirmed in a series of studies which performed in-depth molecular analyses in ICB responder and non-responder tumor tissues (70–72). For instance in human sarcoma, ICB responders are characterized by B cell-rich intratumoral TLS and an immune gene signature related to T cell infiltration and activation, immune checkpoints and expression of Cxcl13 (70). In human melanoma, B cell-enriched TLS confer improved survival and responsiveness to ICB, and also contain naïve and/or memory T cells and an immune signature indicative of enhanced B-T cell interactions and antigen presentation (71, 72). In contrast, T cells in TLS negative melanomas expressed elevated TIM3 and PD-1 levels which may indicate a dysfunctional state (72). Furthermore, RNA-seq analysis of B cell receptors (BCRs) in melanomas showed greater BCR diversity and B cell maturity in ICB responders versus non-responders supporting an active role for B cells in anti-tumor immunity (71). In summary, these studies demonstrate a major role of TLS-associated B cells in antigen presentation, T cell polarization and activation thus placing B cells at the center of TLS function (62, 70–75).

The efficacy of anti-cancer effector T cells is intimately linked to the presence or absence of CD4^+^CD25^+^FoxP3^+^ regulatory T cells (T regs), and interestingly, T reg depletion induces TLS. For instance, in a mouse model of chemically induced fibrosarcoma genetic T reg deletion triggers intratumoral HEV formation, T cell recruitment and tumor control (76, 77). Similarly, T-reg depletion in a model of autochthonous lung adenocarcinoma induces TLS, increases T cell proliferation and DC activation with ensuing tumor control (78).

Overall, current evidence strongly supports a role of intratumoral TLS as priming sites for anti-tumor immunity and prognostic indicators for ICB efficacy. Spontaneous formation of mature and functional TLS in cancer is highly orchestrated and context-dependent; insights into this process will provide exciting opportunities for innovative drug development.

## From Concept to Treatment: Therapeutic Induction of TLS

Experimental TLS induction in animal models provides an important opportunity to study the complex interplay between immune cell populations which foster adaptive anti-cancer immunity. Therapeutic TLS induction in cancer patients holds the promise to advance immunotherapy. Numerous attempts have been made to induce TLS in mouse models, so far with mixed outcomes. For instance, both Ccl21 and LTβR play important roles during peripheral LN development. Early work in a mouse melanoma model indeed found that a recombinant antibody targeting LTα to melanoma cells induced intratumoral HEVs, B and T cell zones, and improved survival (79). In contrast, Ccl21 overexpressing melanoma cells promoted infiltration of suppressive immune cells and cytokines which collectively stimulate tumor growth (80). Thus, to harness TLS therapeutically better mechanistic insights into intratumoral TLS formation are urgently needed.

More recent attempts to induce TLS in mouse tumors have employed sophisticated technologies such as artificial scaffolds, gene engineering, and vaccination strategies. Given the crucial role of LTo cells in the recruitment of LTi during LN development (8, 12), a role of stromal cells as TLS inducers has been widely explored (48, 81–83). For instance, LTα overexpression in a stromal cell line derived from thymus induces lymphoid-like organoids in mice when co-implanted with DCs in a collagenous scaffold (81). Moreover, a collagen sponge with a cocktail of LN-inducing cytokines when implanted under the kidney capsule also initiates formation of artificial LN-like TLS (artTLS) with distinct B/T cell zones, FDC/FRCs and HEVs. Intriguingly, implantation of these sponges into immunodeficient mice generates antibody producing cells following immunization (82), further supporting a role of TLS in adaptive immunity. Similarly, a LN-derived stromal cell line which expresses high levels of the FRC marker podoplanin and chemokines such as Ccl19, Ccl21, Cxcl10 and Cxcl13 - reminiscent of the chemokine gene signature first identified in human colorectal cancer (48) - when implanted subcutaneously in mice also generates TLS (83). Within these TLS, resident T cells were successfully activated into effector T cells by tumor-lysate-pulsed DCs which suppressed the growth of adjacent MC38 colon cancer cells (83).

In gene engineering studies, DCs were generated to produce high levels of T-bet/Tbx21, a transcription factor that drives the development and functionality of immune cells, particularly by producing the key Th1 cytokine IFNγ. T-bet overexpressing DCs also produce high levels of pro-inflammatory cytokines such as TNFα, IL12p40 and IL-36γ, and induce TLS in a mouse colon cancer model; even in the absence of peripheral LNs intratumoral DC-Tbet therapy prolongs survival (84). In contrast, tumor growth control is abolished in IL36R-deficient mice indicating a crucial role of T-bet/IL-36γ in therapeutic TLS induction (84). This is supported by findings in human colon cancer where IL-36γ is highly expressed in M1 macrophages and cells of the vasculature, including vSMCs and HEVs, and correlates with spontaneous TLS formation (85).

In human papilloma virus (HPV) 16-positive cervical cancer, intramuscular vaccination targeting HPV16 E6/E7 antigens induces intratumoral TLS which contain antigen-experienced effector memory T cells (86). Moreover, TLS-rich tumor stroma harbors a typical Th1 gene signature with increased levels of Cxcr3, TBX21, IFNγ and IFNβ.

In human PDAC, T cell infiltration and activation is positively linked to survival in some patients (87, 88), and TLS can be induced following an allogeneic granulocyte-macrophage colony stimulating factor secreting vaccine (GVAX) when given in combination with T reg-depleting cyclophosphamide (89). TLS display a distinct Th17 gene signature, a high T effector to T reg ratio, and serve as a prognostic tool to segregate long term from short term survivors (89). Although this clinical trial provides rare evidence for therapeutic TLS induction in humans, PDAC can harbor spontaneous intratumoral TLS which are linked to better prognosis (41). Interestingly, spontaneous TLS in PDAC are associated with a more mature vascular network that expresses the vascular adhesion molecule VE-Cadherin and is covered by αSMA^+^ pericytes, a mural cell type which wraps around and supports the endothelium (41), suggesting a possible link between TLS formation and stabilized tumor vessels.

## A Potential Link Between Vascular Normalization and TLS Induction

**Figure 2:**
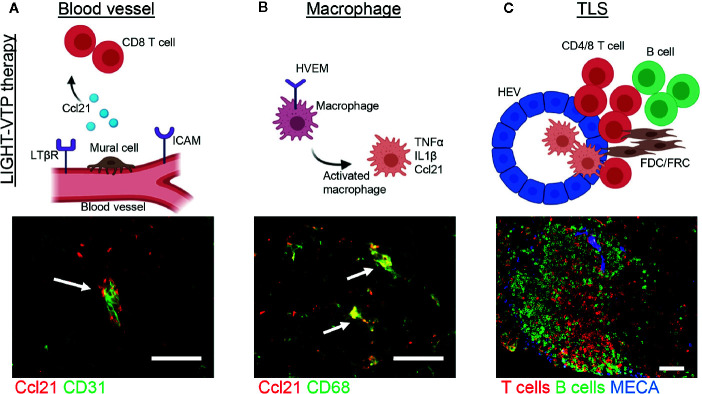
Induction of cancer-associated TLS during LIGHT-VTP therapy. **(A, B)** Treatment of transgenic PNET-bearing mice with bi-weekly i.v. injections of 20 ng LIGHT-VTP specifically targets abnormal angiogenic blood vessels and induces chemokines important for TLS formation (e.g. Ccl21) in **(A)** vascular cells (co-staining of CD31^+^ endothelium in green and Ccl21 in red, overlay in yellow marked by arrow) which attract CD8^+^ T cells, and **(B)** tumor-resident CD68^+^ macrophages which are recruited to the vascular niche (co-staining of CD68 in green and Ccl21 in red, overlay in yellow marked by arrows) and re-programmed to secrete other cytokines such as TNFα and IL1β which in turn attract T/B cells to form TLS (3). **(C)** Adoptive transfer of LIGHT-stimulated macrophages into PNET-bearing mice leads to CD68^+^ macrophage accumulation in the TME and subsequent formation of mature TLS 8 days after transfer. TLS with organized T cell (red) and B cell (green) zones as well as MECA79^+^ HEVs (blue) are depicted. Scale bars 50μm. Images are unpublished microscopic photographs similar to work published in (3). Created with BioRender.com.

T cell infiltration into solid cancers is controlled by the vasculature which co-evolves with an immune-suppressive microenvironment and plays an active part in limiting T cell influx (37, 90–93). In contrast, activating tumor blood vessels to express adhesion molecules such as ICAM and VCAM enables productive endothelial-T cell interactions and fosters effector T cell transmigration (3, 92, 94–97). Moreover, tumor vessel normalization which improves vascular morphology and function lowers hypoxia and indirectly changes the tumor microenvironment to support Th1-driven anti-tumor immunity (98–100). Therefore, compounds which normalize tumor blood vessels and attract T cells may have the capacity to induce intratumoral TLS. Indeed, a fusion compound of the cytokine LIGHT conjugated to a homing peptide (vascular targeting peptide or VTP) which delivers LIGHT specifically to angiogenic tumor vessels is such a reagent (95). LTβR and Herpes virus entry mediator (HVEM) are major LIGHT receptors, expressed in stroma and immune cells, respectively, and thus link LIGHT to LN neogenesis and immune regulation (101–106). Treatment of neuroendocrine pancreatic cancer (PNET) in mice with low dose LIGHT-VTP normalizes blood vessels and induces intratumoral TLS with distinct B and T cell zones and high expression of the T cell attractant Ccl21 in vascular cells as well as macrophages (**Figures 2A, B**) (3, 95). Importantly, the capacity to induce TLS correlates with the degree of vessel normalization and is abolished with high dose LIGHT-VTP which induces vessel death, demonstrating a causal link between vessel normalization and TLS formation (3). Other treatment regimens which are known to normalize tumor vessels in PNET such as low dose anti-vascular endothelial growth factors (VEGF) or anti-angiopoietin-2/anti-VEGF therapies facilitate lymphocyte infiltration but do not induce TLS as monotherapies (107, 108). Similarly, cytokine fusion compounds which deliver for instance TNFα or IFNγ to tumor vessels in PNET induce vessel normalization and/or vessel wall inflammation without TLS formation demonstrating the unique opportunities of targeting LIGHT into the tumor microenvironment (97, 109). Furthermore, intratumoral treatment of melanoma-bearing mice with low dose stimulator of interferon genes (STING) agonist (ADU S-100) normalizes angiogenic blood vessels and upregulates TLS-inducing factors such as Ccl19, Ccl21, LTαβ and LIGHT (110). This induces unstructured HEV-containing lymphocyte aggregates resembling TLS which contain T cells and CD11c^+^ DCs. STING activation enables recruitment of pre-primed peripheral T cells and expansion of unique T cell clonotypes in the TME thus further supporting the benefits of reagents with dual capacity to induce vessel normalization and intratumoral priming. Nevertheless, the anti-tumor effects of LIGHT-VTP or STING monotherapies are modest, and the clinical relevance of these reagents lies in increasing the potency of current immunotherapies (3, 110).

## TLS and Immunotherapy

Immunotherapies which boost the host’s intrinsic immunity such as anti-cancer vaccines and ICBs have dramatically changed clinical oncology. However, based on the increasing number of drug combination trials, ICB therapies will be predictably more effective in combination with other therapies such as TLS induction (7, 111).

The presence of spontaneously arising B cell-rich TLS within cancers has recently been shown to predict the response to ICB in patients with melanoma, soft-tissue sarcoma and renal cell carcinoma (see above) (70–72). In addition, a retrospective analysis of human lung cancer samples identified PD-1^hi^ expressing CD8^+^ T cells within TLS to predict response to PD-1 blockade (112). These proliferating PD-1^hi^ T cells were highly tumor-reactive, secreted Cxcl13, and are thus potential drivers of TLS formation (112). Similarly, non-small cell lung carcinoma biopsies from PD-1 blockade responders are enriched in TLS and mature B cells (113). Furthermore, patients with desmoplastic melanoma, a subtype of melanoma with dense fibroblastic stroma and high frequency of TLS, respond particularly well to PD-1 blockade compared to other advanced forms of melanoma (114). Although the correlation of TLS frequency and patient responsiveness in retrospective studies might be biased, collectively these studies support the notion that TLS induction prior to ICB is beneficial and will improve response rates to immunotherapy.

Strong evidence for beneficial TME-immune stimulating combination therapies also comes from animal studies. For instance, experimental induction of TLS with LIGHT-VTP therapy renders PNET and Lewis lung carcinoma (LLC) sensitive to ICB targeting cytotoxic T-lymphocyte-associated protein 4 (CTLA-4) and PD-1. The combined treatment induces intratumoral activation of cytotoxic T cells with ensuing survival benefits which can be further improved when combined with anti-cancer vaccination. Notably, neither vaccination, ICB or a combination thereof match the survival outcome achieved with LIGHT-VTP combination treatment (3). In mouse breast cancer, PNET, and glioblastoma (GBM), VEGF inhibition renders tumors susceptible to anti-PD-L1 therapy. The combination treatment of anti-VEGF and anti-PD-L1 activates intratumoral DCs and T cells and reaches maximal efficacy when combined with agonistic LTRβ antibodies; this triple treatment induces HEV^+^ immune clusters even in highly therapy-resistant GBM (115). In the same GBM tumor model, LIGHT-VTP treatment in combination with anti-VEGF and anti-PD-L1 is even more effective than agonistic LTβR antibodies, and generates an abundance of intratumoral HEV^+^ TLS and granzyme B^+^ (GrzB) CD8^+^ effector T cells (116). This highlights the importance of LTβR signaling for TLS combination immune therapies but also the potential involvement of other pathways since LIGHT activates cells within the tumor microenvironment through multiple receptors including LTβR and HVEM.

Overall, there is already strong evidence that intratumoral TLS are an important prognostic tool for immunotherapies (70–72). However, beyond risk stratification, inducing TLS in combination with ICB generates a synergism which is likely to promote lymphocyte infiltration, intratumoral activation and immune rejection, in particular in immune-deserted or “cold” tumors. Given the significant toxicities of ICB as observed in recent combination trials of Nivolumab and Ipilimumab (117, 118), the presence of TLS may be helpful to select patients who will benefit most from ICB. In addition, TLS/ICB combination therapies could contribute to more effective anti-tumor responses with lower ICB doses. In this context, a preliminary study of low dose Nivolumab and Ipilimumab combined with IL-2 and hyperthermia treatment shows similar overall response rates when compared to high dose ICB with significantly lower overall toxicity (119). This indicates an exciting possibility to lower ICB doses when used in combination with other immune stimulating reagents.

## Searching for the Instigator(s) in Cancer-Associated TLS

**Figure 3:**
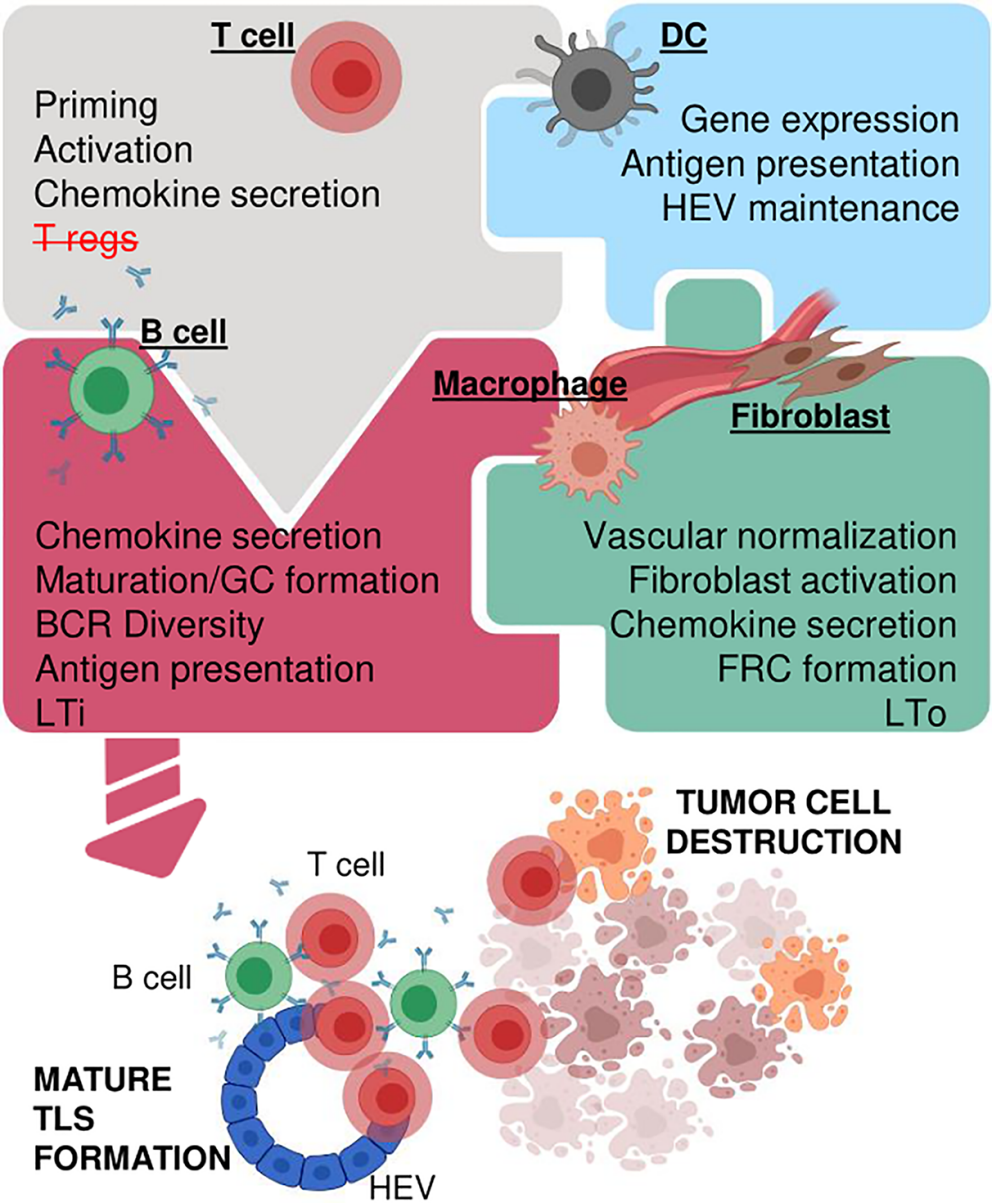
Concepts for creating functional TLS in cancer. Multiple immune and stromal cell types orchestrate TLS formation potentially involving activated T cells and/or depletion of T regs (upper left), DCs which express TLS supporting cytokines/chemokines, maintain HEVs and promote antigen presentation (upper right), mature B cells which produce antibodies, enhance antigen presentation, act as LTi cells (lower left), and stromal cells such as fibroblasts, macrophages and vascular cells which can act as LTo, secrete chemotactic cytokines/chemokines and/or provide structural support (lower right). Some or all cell types may be necessary in an intricate network of simultaneous or consecutive interactions to generate mature TLS which in turn enhance immunotherapy and tumor destruction. Created with BioRender.com.

Much like LN neogenesis, formation of cancer-associated TLS presumably involves a network of stromal and immune cells linked by multiple cytokines/chemokines. However, mechanistic insights into this process are rudimentary. Since these interactions are precisely orchestrated in a 3D environment *in vitro* studies are challenging. Nevertheless, some cell types and cytokines/chemokines by virtue of their crucial role in experimental systems and presence in human TLS^+^ cancer tissue deserve further consideration (**Figure 3**).

### Non-Hematopoietic Stromal Cells: Blood Vessels and Fibroblasts

Tumor vasculature and TLS formation are intimately linked (3, 4, 110). For instance, LIGHT-VTP in mouse PNET increases the expression of Ccl21 in the vascular bed and in CD68^+^ tumor-resident macrophages associated with TLS (**Figures 2A, B**) (3). Moreover, a 3D scaffold environment and slow interstitial flow are essential for Ccl21 expression by LN-derived FRCs both *in vitro* and *in vivo*; without lymph flow Ccl21 expression is not detectable suggesting that fluid flow dynamics may regulate Ccl21 expression (120). It is therefore interesting to speculate that modulation of blood flow dynamics and transport of cytokines/chemokines during tumor blood vessel normalization may regulate Ccl21 expression levels in the vascular bed, and thus TLS formation *in vivo*.

Cancer associated fibroblasts (CAFs) form a large part of the tumor microenvironment, reduce fluid flow by increasing tumor stiffness, and support tumor-promoting inflammation (121). Thus, modulation of CAFs can enhance anti-cancer immunotherapy (121, 122) and potentially support TLS formation. More recently, a crucial role for CAFs as LTo and effector CD8^+^ T cells/B cells as LTi was delineated in an intraperitoneal melanoma model of spontaneous TLS formation (4). Therein, effector T cells recruit FAP^-^ podoplanin^+^ fibroblasts to HEVs where they differentiate into Cxcl13 secreting FRCs *via* TNFR signaling, similar to previous models of chronic inflammation (26, 27). This in turn promotes recruitment and proliferation of LTα_1_β_2_ secreting B cells which further stimulate TLS formation in a positive feedback loop (4). In human and mouse lung cancer, Ccl19 producing fibroblastic stromal cells (FSC) correlate with increased CD8^+^ T cell infiltration and tumor growth control. Although TLS formation was not examined in this study, Ccl19-expressing FSCs reside in peri-vascular niches within LLC tumors and T cell recruitment is impaired upon Ccl19 gene deletion suggesting an early role of FSCs in forming immune-stimulating stromal niches (123). Collectively these studies support the notion that vascular cells and fibroblasts are important mediators of TLS neogenesis in cancer (4, 38, 94).

### Hematopoietic Stromal Cells: Macrophages

Monocytes/macrophages are a major component of tumor stroma (124). In a hypoxic tumor environment, macrophages are immunosuppressive and support tumor growth. However, their phenotype is highly dynamic and macrophage “re-education” can support immunotherapy (125). In the context of TLS neogenesis, M1 macrophages can produce chemokines similar to those detected in TLS^+^ human cancers, including Ccl21 and TNFα (3, 126). Furthermore, *ex vivo* LIGHT-stimulated macrophages in contrast to control macrophages when adoptively transferred into tumor-bearing mice are necessary and sufficient to induce intratumoral TLS in a T cell-dependent manner (**Figure 2C**) (3). In addition to Ccl21, these LIGHT-stimulated macrophages also express high levels of TNFα (**Figure 2B**) which is a key driver of inflammation-induced TLS formation in mice (21, 27). It is therefore possible that LIGHT-stimulated macrophages drive TLS formation *via* the TNFα/TNFR signaling pathway which has so far not been investigated. Whilst the importance of macrophages during TLS formation in cancer is understudied, robust data in inflammatory disease support their importance in TLS neogenesis (21, 27, 29), warranting further investigations in cancer.

### Hematopoietic Stromal Cells: DCs

LTα/LTβ producing CD11c^+^ DCs play a critical role in regulating lymphocyte trafficking and maintaining HEV phenotype and function in adult mouse LNs (30, 32), and are involved in TLS formation during chronic inflammation (30–35). In human tumors, DCs are a major source of LTβ and their density correlates with HEV formation and favorable clinical outcome in breast cancer (60). Similarly, in primary human lung and ovarian cancers the number of mature DCs correlates with the degree of CD8^+^ T cell infiltration, anti-tumor cytotoxicity and survival (42, 127). Furthermore, immune-stimulating and vascular normalization therapies in mice increase intratumoral CD11c^+^ DCs coinciding with the formation of lymphocyte aggregates and HEVs (110, 115). Treatment of B16 melanoma with low-dose STING agonist, for instance polarizes DCs to produce TLS-inducing cytokines such as LTα, IL36β and TNFα (110), implicating mature DCs in TLS neogenesis. Overall, mechanistic tumor data are still sparse; plasticity of myeloid cells as well as shared marker expression in myeloid cell and DC populations complicate interpretation of the data. Further analysis of stromal innate immune cells such as monocytes/macrophage and DCs as initiators of cancer-associated TLS is therefore warranted.

## Conclusions

Although immunotherapy has shown unprecedented success in some cancer patients and tumor types, the challenge ahead lies in improving the outcome for non-responsive patients. TLS as prognostic markers for improved patient outcomes have long been recognized (7). However, only recently have mature TLS been shown to predict ICB success in patients (70–72). It is imperative to now develop strategies to increase TLS frequency and/or maturation in cancers where they naturally occur. This may be achieved by providing further innate immune stimulation as demonstrated for instance with STING agonist treatment (110). Induction of *de novo* TLS formation holds great therapeutic potential to overcome intrinsic immune inhibitory mechanisms within the TME and render non-responsive, immune “cold” tumors susceptible for ICB. However, the orchestration of mature immune-supportive TLS formation in cancer is complex and involves multiple cellular compartments and cytokines/chemokines; this process may also be tumor type-dependent. Emerging mechanistic insight from mouse tumors demonstrate potential LTi roles for anti-tumor effectors such as T and surprisingly B cells which requires re-definition of the role of B cells in TLS and cancer (4). Therapeutic vessel normalization which enables lymphocyte infiltration into tumors may also promote access of these LTi into the TME for more effective TLS priming (3, 110). Furthermore, intratumoral stromal cell types such a monocytes/macrophages and fibroblasts are strong candidates for LTo cells which when reprogrammed in permissive tumor “niches” can drive TLS formation (3, 4). In this context, TNFR in addition to LTβR signaling may prove crucial for tumor-associated TLS formation as opposed to primarily LTβR driven processes as seen during peripheral LN development. Overall, improving existing TLS function or priming *de novo* TLS formation in cancer to maximize ICB efficacy holds the potential to induce more durable anti-tumor immune responses in a higher percentage of cancer patients and warrants urgent investigation.

## Author Contributions

AJ-P designed the figures. AJ-P and RG planned, constructed and wrote the paper. All authors contributed to the article and approved the submitted version.

## Funding

This work was supported by the National Health and Medical Research Council of Australia (APP1157240, APP2001120), Cancer Council Western Australia, Cancer Research Institute Clinic and Laboratory Integration Program (CLIP), Worldwide Cancer Research and a Woodside Energy Fellowship (to RG). The funder was not involved in the study design, collection, analysis, interpretation of data, the writing of this article or the decision to submit it for publication.

## Conflict of Interest

The authors declare that the research was conducted in the absence of any commercial or financial relationships that could be construed as a potential conflict of interest.
